# A Novel Combination of Withaferin A and Sulforaphane Inhibits Epigenetic Machinery, Cellular Viability and Induces Apoptosis of Breast Cancer Cells

**DOI:** 10.3390/ijms18051092

**Published:** 2017-05-19

**Authors:** Kendra J. Royston, Neha Udayakumar, Kayla Lewis, Trygve O. Tollefsbol

**Affiliations:** 1 Department of Biology, University of Alabama at Birmingham, 1300 University Boulevard, Birmingham, AL 35294, USA; kendroy@uab.edu (K.J.R.); nehau@uab.edu (N.U.); klalewis@uab.edu (K.L.); 2 Comprehensive Cancer Center, University of Alabama Birmingham, 1802 6th Avenue South, Birmingham, AL 35294, USA; 3 Comprehensive Center for Healthy Aging, University of Alabama Birmingham, 1530 3rd Avenue South, Birmingham, AL 35294, USA; 4 Nutrition Obesity Research Center, University of Alabama Birmingham, 1675 University Boulevard, Birmingham, AL 35294, USA; 5 Comprehensive Diabetes Center, University of Alabama Birmingham, 1825 University Boulevard, Birmingham, AL 35294, USA

**Keywords:** sulforaphane, withaferin, breast cancer, histone deacetylase1 (HDAC1), DNA methyltransferases (DNMTs), chemoprevention, epigenetics

## Abstract

With cancer often classified as a disease that has an important epigenetic component, natural compounds that have the ability to regulate the epigenome become ideal candidates for study. Humans have a complex diet, which illustrates the need to elucidate the mechanisms of interaction between these bioactive compounds in combination. The natural compounds withaferin A (WA), from the Indian winter cherry, and sulforaphane (SFN), from cruciferous vegetables, have numerous anti-cancer effects and some report their ability to regulate epigenetic processes. Our study is the first to investigate the combinatorial effects of low physiologically achievable concentrations of WA and SFN on breast cancer cell proliferation, histone deacetylase1 (HDAC1) and DNA methyltransferases (DNMTs). No adverse effects were observed on control cells at optimal concentrations. There was synergistic inhibition of cellular viability in MCF-7 cells and a greater induction of apoptosis with the combinatorial approach than with either compound administered alone in both MDA-MB-231 and MCF-7 cells. HDAC expression was down-regulated at multiple levels. Lastly, we determined the combined effects of these bioactive compounds on the pro-apoptotic *BAX* and anti-apoptotic *BCL-2* and found decreases in *BCL-2* and increases in *BAX*. Taken together, our findings demonstrate the ability of low concentrations of combinatorial WA and SFN to promote cancer cell death and regulate key epigenetic modifiers in human breast cancer cells.

## 1. Introduction

Epigenetics is the study of changes in gene expression caused by mechanisms other than changes in the underlying DNA sequence. Many studies have taken an epigenetic approach to cancer prevention by focusing on the modulation of the expression of key epigenetically controlled genes [1]. It is known that several cancers are characterized by an overexpression of histone deacetylases (HDACs) and DNA methyltransferases (DNMTs). Each of these epigenetic enzymes has varying roles. The inhibition and regulation of these enzymes, as well as the genes that control their expression, are at least partially responsible for decreased cell viability and regulation of tumor suppressor genes in several cancer types [2,3,4]. Due to the promising role of the inhibition of epigenetic modifiers in cancer cell death, chemotherapies with epigenetic targets have been US Food and Drug Administration (FDA)-approved and are being used in the clinical setting [5].

Breast cancer, one of the leading causes of death in women in the United States, has an incidence rate of more than 200,000 new cases and a mortality rate of about 40,000 women per year [6]. Numerous investigations have been launched with the intent to better understand novel approaches to enhance current chemotherapies as well as preventing the acquisition of the disease through the consumption of dietary compounds, which may be responsible for epigenetic modifications to the genome. Recently, Esmaeili reported that epigallocatechin gallate (EGCG), a component of green tea, is responsible for the reversal of chemoresistance in breast cancer cells [7]. Moreover, our studies have indicated that genistein, a soybean isoflavone, is instrumental in the reactivation of estrogen receptor α (ERα) in triple-negative breast cancer cells, which enhanced the efficacy of hormone therapy in these cells [8]. The regulation of DNMTs and HDACs was shown to be an important factor in ERα conversion in these cells. In addition, sulforaphane (SFN) can be effective in the inhibition of several different cancer types in part through its ability to serve as an epigenetic modifier [9,10,11,12,13].

### Sulforaphane (SFN) and Withaferin A (WA)

SFN is an isothiocyanate found in cruciferous vegetables that has shown promising results in chemoprevention and is of high interest due to its role in HDAC inhibition [14,15]. This dietary bioactive compound promotes apoptosis and prevents the continued proliferation of breast cancer cells through various mechanisms. For example, SFN can work well in conjunction with other compounds, thereby increasing the efficacy of programmed cell death and the regulation of epigenetic processes within many different cell lines [16,17]. Our lab has conducted a number of studies with regard to the epigenetic impact of SFN in conjunction with other dietary compounds in cancer and their ability to promote cancer cell death. A review of the literature led to the curiosity of how SFN interacts with withaferin A, a relatively novel compound in the field of cancer epigenetics. We aimed to determine if there would be an enhanced efficacy in the inhibition of key epigenetic modifiers that are known to affect cell cycle progression in several different cancer types. Withaferin A, a withanaloid isolated from a winter cherry prevalent in India, has promising roles in cancer prevention and therapy. The plant from which this compound is derived has roots that have been used medicinally for years by the indigenous population due to its wound healing properties. Withaferin A (WA) is a steroidal lactone that can lead to decreased cellular proliferation and viability in certain cancer cell lines, regulate inflammatory pathways, and is an inducer of apoptosis, all of which have piqued the interest in use of this compound as a potential chemotherapeutic agent [18,19,20,21]. In contrast, however, less is known about the epigenetic roles of WA, although some studies have found that it behaves as a DNMT inhibitor [22]. This compound has also received much acclaim due to its promise in the inhibitory effects of angiogenesis, which is a fundamental step in the formation of malignant tumors [23]. Thaiparambil et al. have shown WA to be effective in the inhibition of breast cancer invasion and metastasis through its ability to induce vimentin disassembly [18]. It may be possible that WA has the ability to prevent the malignant behavior of tumors while lessening the incidence of carcinogenic fatality.

In this study we aimed to investigate the impact of combinatorial SFN and WA on MCF-7 estrogen receptor-positive (ER (+)) and MDA-MB-231 ER (−) breast cancer cell proliferation in conjunction with their role in the epigenetic gene expression of DNMT1, DNMT3A, DNMT3B and HDAC1. The present study is the first to show changes in the expression of epigenetic modifiers using these two compounds in combination at such low concentrations.

## 2. Results

### 2.1. Combinatorial WA and SFN Promote Cell Death

**Figure 1:**
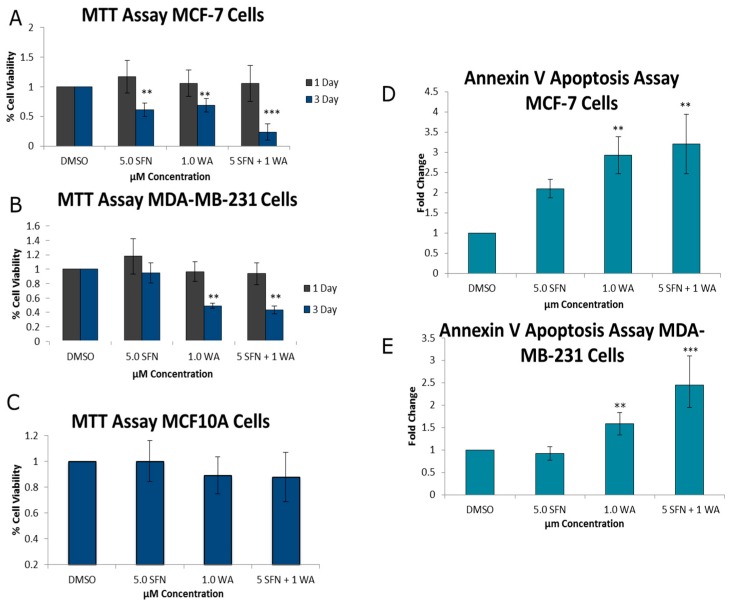
Combinatorial withaferin A (WA) and sulforaphane (SFN) decrease cellular viability and promotes apoptosis in breast cancer cells. (**A**) Assays with 3-(4,5-dimethylthiazol-2-yl)-diphenyl tetrazolium bromide (MTT) of MCF-7 breast cancer cells were performed using either 5.0 µM SFN, 1.0 µM WA, or both compounds at the indicated concentrations for a period of either 1 or 3 days; (**B**) MTT assays were performed on MDA-MB-231 cells for 1 or 3 days at the indicated concentrations; (**C**) MTT assay was performed on MCF10A cells for 3 days using the same concentrations mentioned previously; (**D**) Annexin V Apoptosis assay employing FACS analysis was completed on MCF-7 cells using 5.0 µM SFN, 1.0 µM WA, or 5.0 µM SFN + 1.0 µM WA for 3 days; (**E**) Apoptosis assay was completed on MDA-MB-231 breast cancer cells using 3-day treatments at the indicated concentrations of SFN, WA or both compounds (** *p* < 0.01, *** *p* < 0.001). The results represent three separately cultured experimental replicates. Numbers on the X-axis indicate compound concentrations.

**Figure 2:**
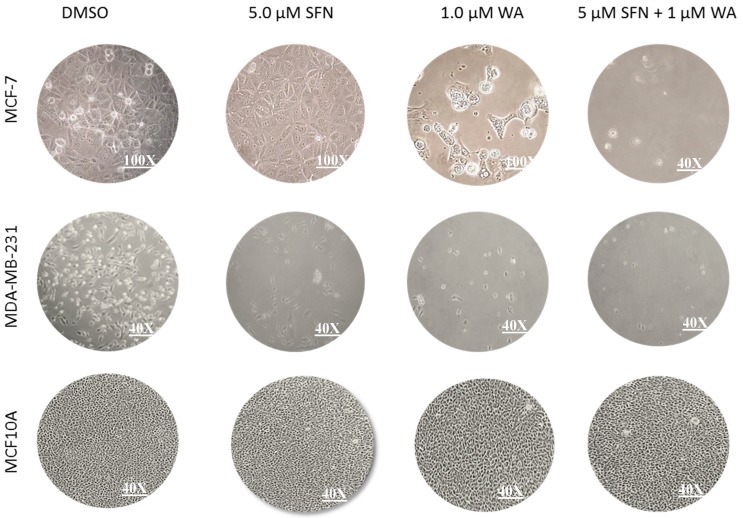
Combinatorial WA and SFN promote increases in surface area of breast cancer cells. MCF-7 and MDA-MB-231 breast cancer cells as well as the non-cancerous MCF10A cells were treated with predetermined optimized concentrations of 5.0 μM SFN or 1.0 μM WA singly and both compounds at the same concentrations for 3 days where DMSO serves as the control. Photographs were taken on the fifth day of culture.

As seen in Figure 1A,B, treatments were administered for one- and three-day intervals. At one day, cancer cells were unaffected by treatments; however, after three days each of the compounds administered individually, cancer cell death was induced as indicated through decreases in cell viability with 3-(4,5-dimethylthiazol-2-yl)-diphenyl tetrazolium bromide (MTT) analysis and increases in the induction of apoptosis. However, there was a greater impact when used in combination. Both cell lines show these compounds to be effective in promoting cancer cell death after three days (Figure 1D,E). No significant effects of these compounds administered singly or in combination were observed after three days (Figure 1C) on control MCF10A cells, indicating the relative safety of these compounds at the low concentrations that were employed. As evidenced through cell density analysis in Figure 2, both MCF-7 and MDA-MB-231 breast cancer cell lines show an increase in surface area with the incorporation of the predetermined optimal concentrations of 1.0 µM WA and 5.0 µM SFN in comparison to the MCF10A non-cancerous control cells. Moreover, the combination of administered 1.0 µM WA and 5.0 µM SFN was more effective than either of the compounds acting alone. Using CompuSyn software analysis [24] we observed a synergistic effect with our combined concentrations in the MCF-7 ER (+) cells and an additive effect with combinatorial WA and SFN in MDA-MB-231 ER (−) breast cancer cells (Table 1).

Using the software CompuSyn Version 1.0 by Ting Chao Chou and Nick Martin to determine combination index (CI) [24], we were able to show synergy (CI < 1) with combinatorial WA and SFN treatments of MCF-7 cells and an additive effect (CI = 1) for the MDA-MB-231 breast cancer cells.

### 2.2. Combinatorial WA and SFN Administration Decreases HDAC Expression and Promotes Varying Changes in DNMT Expression

**Figure 3:**
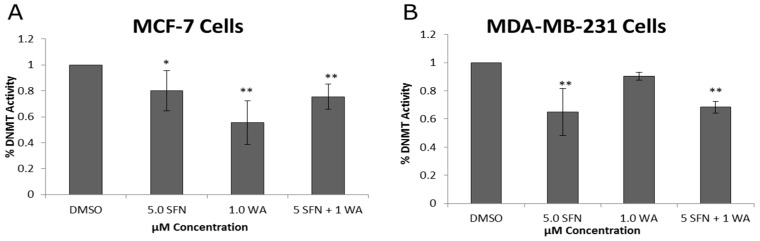
Combinatorial WA and SFN affect enzymatic activity of DNA methyltransferases (DNMTs). (**A**) DNMT activity assays were conducted using nuclear extracts that were prepared after 3-day treatments with the indicated concentrations in MCF-7 cells; (**B**) DNMT activity was assessed in the same way using MDA-MB-231 cells. Depicted results are the means of four separately cultured experiments. (* *p* < 0.05, ** *p* < 0.01)

In an effort to understand some of the mechanisms underlying our observations we next sought to determine any changes in the expression of known epigenetic modifiers, DNMTs and HDACs in the treated cells. In Figure 3 we demonstrate decreases in enzymatic activity of DNMTs in both cell lines. The combination treatment of SFN and WA in MCF-7 cells is more effective in the inhibition of DNMT activity than singly administered SFN but not WA, and in MDA-MB-231 cells the combination treatment effect was highly significant, more so than with the singly administered WA. Clearly these compounds are capable of modulating DNMTs activity in at least two commonly used cell types of breast cancer.

**Figure 4:**
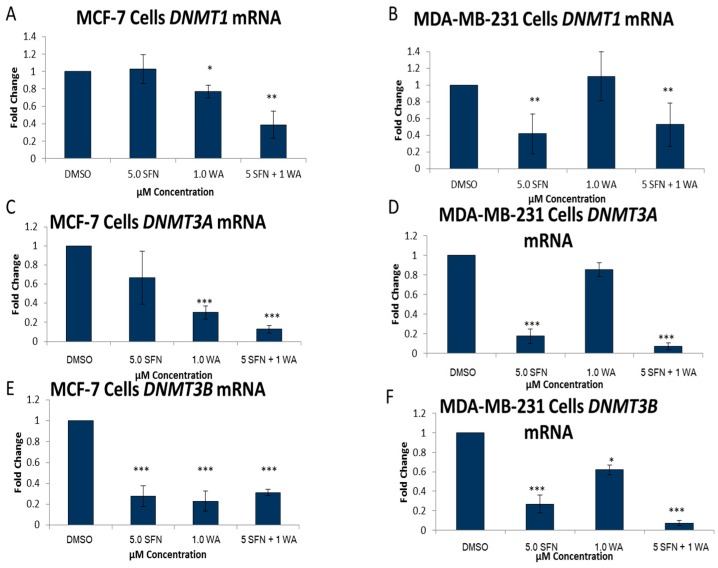
Combinatorial WA and SFN induce changes in the mRNA expression of *DNMTs.* (**A**) qRT-PCR was completed using MCF-7 cells after 3-day treatments of the indicated compounds was conducted using *DNMT1* forward and reverse primers (*n* = 3). *GAPDH* was used for comparison; (**B**) The same was done in MDA-MB-231 cells (*n* = 4); (**C**) qRT-PCR was performed in MCF-7 cells using *DNMT3A* primers. (*n* = 3); (**D**) qRT-PCR was performed in MDA-MB-231 cells using *DNMT3A* primers (*n* = 3); (**E**) *DNMT3B* mRNA expression was determined in MCF-7 cells; (**F**) *DNMT3B* mRNA expression in MDA-MB-231 cells (*n* = 3). (* *p* < 0.05, ** *p* < 0.01, *** *p* < 0.001)

In an effort to examine specific DNMTs we performed quantitative real time PCR on *DNMT1*, *DNMT3A* and *DNMT3B* as seen in Figure 4. WA decreased *DNMT1* mRNA expression in MCF-7 cells, and this was more pronounced with the combinatorial treatments of SFN + WA (Figure 4A). In MDA-MB-231 cells the combinatorial treatment also led to a significant decrease in DNMT1 expression with varying effects on *DNMT1* expression by the compounds administered singly (Figure 4B). Due to these varying effects on *DNMT1* and the results from the DNMT activity analysis we decided to determine if there were any changes in *DNMT3A* and *DNMT3B*. It can be noted that *DNMT3A* and *DNMT3B* mRNA expression is down-regulated in an extremely significant manner in both cell lines (Figure 4C–F).

**Figure 5:**
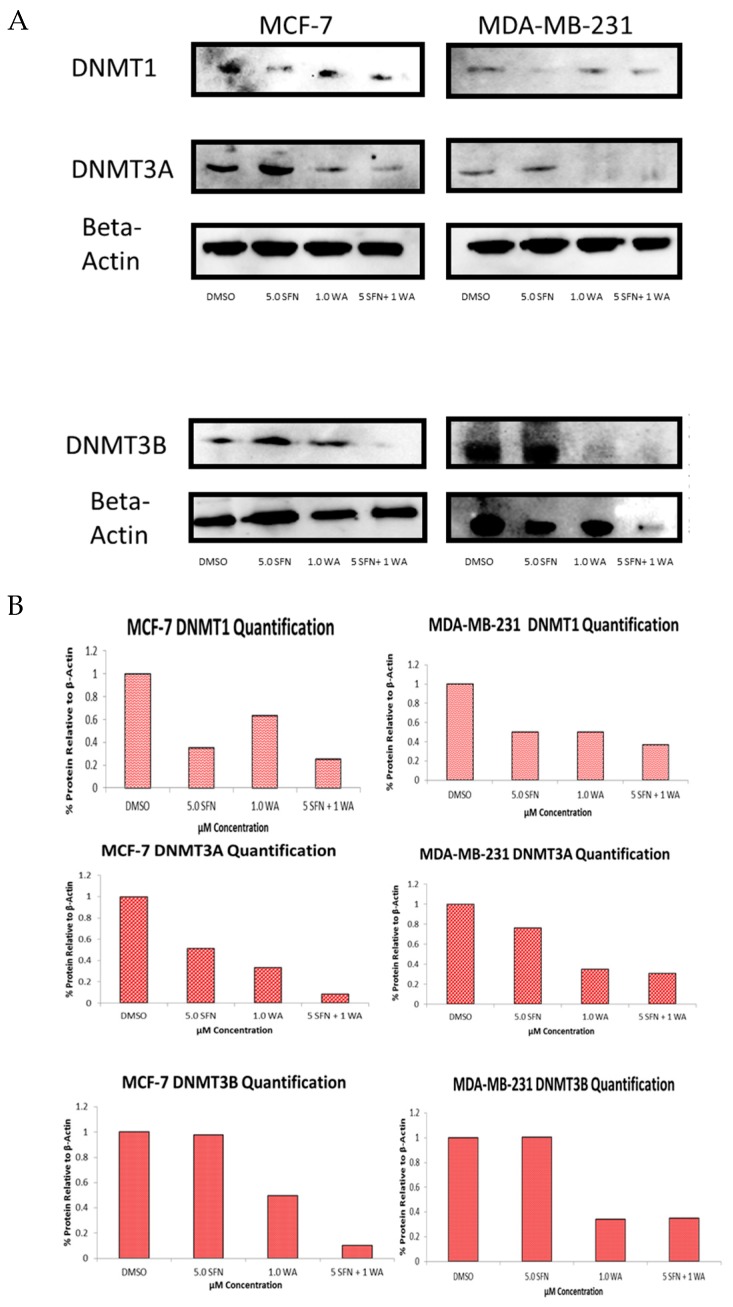
Combinatorial WA and SFN promote decreases in DNMT protein expression. (**A**) Representative images of the protein expression for DNMT1, DNMT3A and DNMT3B in both MCF-7 and MDA-MB-231 breast cancer cells are shown. Western blots were completed after 3-day treatments of the indicated concentrations and probed with the corresponding antibodies; (**B**) Quantification was performed using the averages of multiple blots using ImageJ software (National Institute of Health, Bethesda, MD, USA).

**Figure 6:**
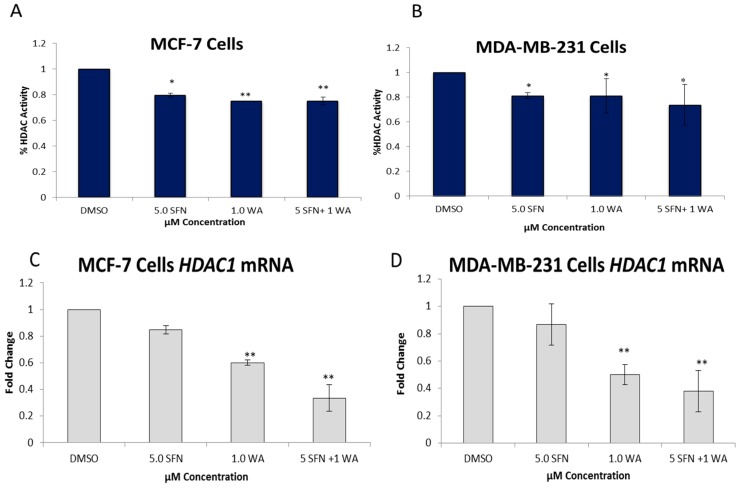
Combinatorial WA and SFN down-regulate histone deacetylase1 (HDAC) activity and mRNA expression. (**A**) HDAC activity/ inhibition assays were performed using nuclear extracts from 3-day treatments of MCF-7 cells at the indicated concentrations; (**B**) MDA-MB-231 cell HDAC activity assays were performed using the same methodology described above; (**C**) qRT-PCR was completed to determine the mRNA expression of *HDAC1* in MCF-7 cells; (**D**) *HDAC1* mRNA expression is shown in MDA-MB-231 cells. The results represent the means of three separately cultured experimental replicates. (* *p* < 0.05, ** *p* < 0.01)

**Figure 7:**
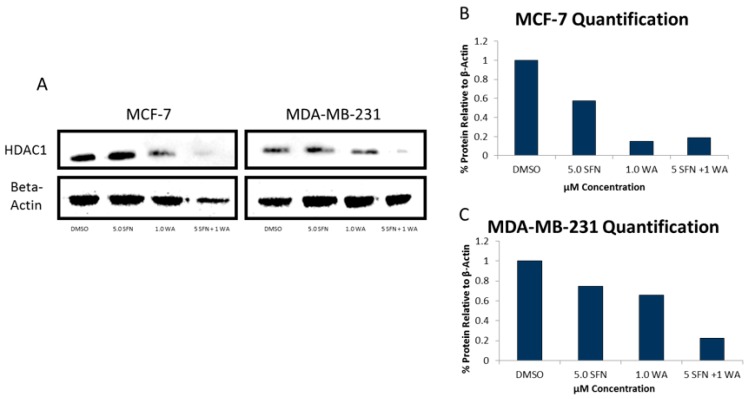
Combinatorial WA and SFN change the expression of HDAC1 at the protein level. (**A**) MCF-7 cells (**left**) and MDA-MB-231 cells (**right**) were treated for 3 days at the indicated concentrations and images are representative. Protein was extracted and used to perform western blot analysis of HDAC1; (**B**) ImageJ was used to quantify results. The results are presented in comparison to β-actin as indicated via the bar graph for MCF-7 cells; (**C**) HDAC1 protein quantification was completed in MDA-MB-231 cells.

We also determined the protein expression of each of these DNMTs as shown in Figure 5 and show that the SFN + WA treatments were effective in the inhibition of DNMT1, DNMT3A and DNMT3B in comparison to the dimethyl sulfoxide (DMSO) control. Next, we sought to determine the effects of WA and SFN on HDACs and found significant decreases in HDAC activity in both cell lines with the incorporation of our compounds (Figure 6); however, the MDA-MB-231 cells do not show greater significance after combinatorial treatment (Figure 6B). A downward trend in the mRNA expression fold change of HDAC1 was observed at all tested concentrations in both cell lines (Figure 6C,D). This was highly significant for the combinatorial treatments of SFN + WA. In Figure 7 it can be noted that western blot analysis revealed that HDAC1 was down-regulated post-translationally with the incorporation of the selected compounds and that this effect was most apparent in the combination treatments in both MCF-7 and MDA-MB-231 breast cancer cells.

### 2.3. Combinatorial WA and SFN Induce Changes in BAX and BCL-2

**Figure 8:**
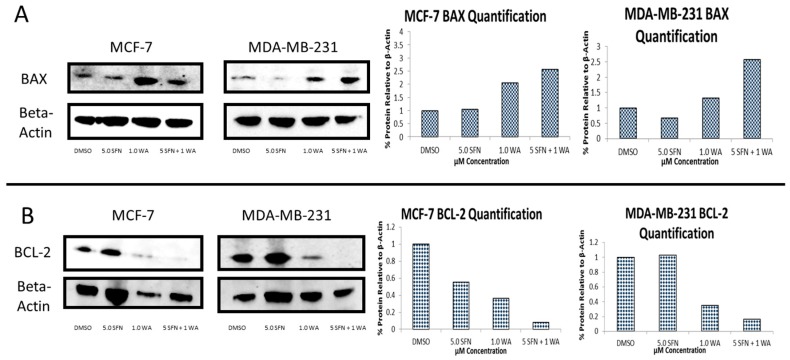
Combinatorial WA and SFN induce changes in the protein expression of the pro-apoptotic BCL-2 and anti-apoptotic BAX. (**A**) BAX protein expression in both MCF-7 and MDA-MB-231 cells is induced with the incorporation of the natural compounds. Image is representative and quantification is indicative of the averages of three different blots; (**B**) BCL-2 protein expression in both MCF-7 and MDA-MB-231 is reduced with the incorporation of the natural compounds. Image is representative and quantification is indicative of the averages of three different blots.

**Figure 9:**
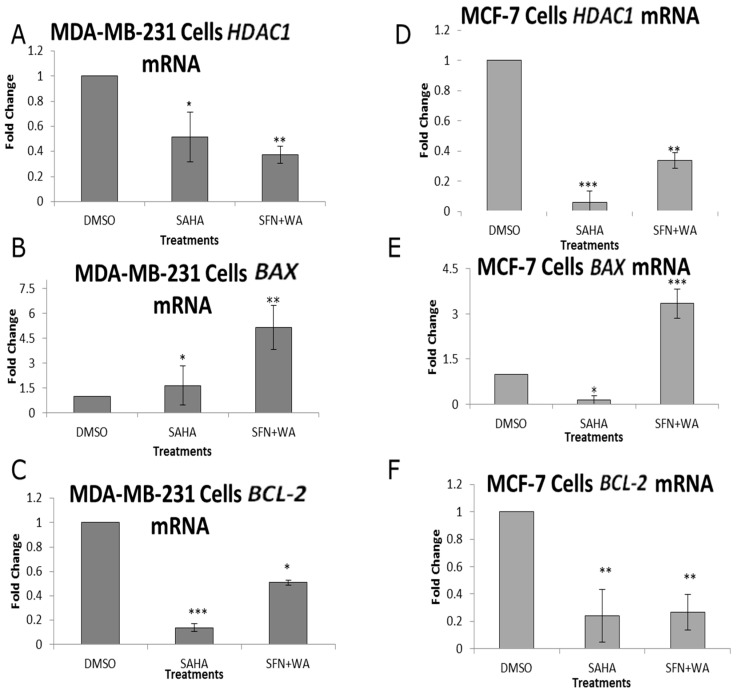
Suberoylanilide hydroxamic acid (SAHA) and combinatorial SFN + WA promote changes in apoptotic genes at the mRNA level. (**A**) qRT-PCR was used to determine the mRNA expression of HDAC1 in MDA-MB-231 triple-negative breast cancer cells in comparison to the 3.0 µM optimal concentration of SAHA. SFN and WA concentrations are 5.0 and 1.0 µM respectively; (**B**) mRNA expression of the pro-apoptotic *BAX* is shown in MDA-MB-231 cells; (**C**) qRT-PCR shows the expression of the anti-apoptotic *BCL-2* in MDA-MB-231 cells; (**D**) HDAC1 mRNA expression in MCF-7 cells shows changes with the incorporation of the chosen compounds; (**E**) mRNA expression of the pro-apoptotic gene BAX is upregulated by combinatorial SFN and WA; (**F**) The anti-apoptotic gene *BCL-2* shows a downward progression in MCF-7 cells with the incorporation of the indicated drugs and compounds (*n* = 3: SEM, * *p* < 0.05, ** *p* < 0.01, *** *p* < 0.001). SEM: standard error of mean.

BAX and BCL-2 have been shown to be inversely associated with one another. Due to the ability of combinatorial WA and SFN to promote apoptosis in both MCF-7 and MDA-MB-231 cells, we sought to determine the expression of both BAX and BCL-2 (Figure 8). Our results show BAX expression to be induced, whereas BCL-2 expression is inhibited. The consistent downward trends found in HDAC expression in both cell lines led us to believe that combinatorial WA and SFN decrease cell viability and promote apoptosis in part through their ability to inhibit HDAC1. In Figure S1 we demonstrate the ability of suberoylanilide hydroxamic acid (SAHA) to decrease cell proliferation in both MCF-7 and MDA-MB-231 cells as was shown with combinatorial WA and SFN (Figure 1). SAHA is an HDAC inhibitor that is clinically approved and marketed as Vorinostat for the treatment of cutaneous T-cell lymphoma (CTCL). Because this is a synthetic compound, we expected to see some reduction in viability in our noncancerous control MCF10A cells. Using MCF-7 and MDA-MB-231 cells there is a significant decrease in cellular viability beginning at 2 µM of SAHA and continuing through to 7 µM of SAHA (Figure S1). We show in Figure 9 that these compounds, in a similar fashion to SAHA, affect both apoptotic genes, *BAX* and *BCL-2*, while repressing *HDAC1*. It can be noted that while SAHA inhibits *HDAC1* expression, combinatorial WA and SFN are more effective in the MDA-MB-231 cells (Figure 9A). In contrast, SAHA is more effective than SFN + WA in the inhibition of *HDAC1* expression in MCF-7 cells (Figure 9D). Figure 9B, E show SFN + WA to be effective inducers of *BAX* expression to a greater degree than SAHA in MDA-MB-231 cells (Figure 9B); *BAX* expression is decreased with SAHA treatment in MCF-7 cells (Figure 9E). Figure 9C demonstrates the ability of SAHA and SFN + WA to decrease *BCL-2* expression in the ER (−) MDA-MB-231 cells. The same is shown in Figure 9F for the ER (+) MCF-7 cells.

## 3. Discussion

For the first time we report the epigenetic effects of combinatorial WA and SFN in any cancer type. This study is of particular interest due to the increasing awareness of the effects of dietary compounds on epigenetic changes in cancer. We report that combinatorial WA and SFN were more effective than either compound alone in decreasing cellular viability and promoting apoptosis in both MCF-7 ER (+) and MDA-MB-231 ER (−) breast cancer cells at relatively low concentrations (Figure 1). Synergy from this unique approach using combined WA and SFN in cancer cells was detected in MCF-7 cells and we found additive effects in the MDA-MB-231 cells (Table 1). Previous studies showed SFN to be an effective HDAC inhibitor. Specifically, Clarke et al. reported SFN to be an effective inhibitor of several class I and II HDACs. In their study they compared normal prostate cells with cancerous and hyperplastic prostate cells and demonstrated a selective induction of cell cycle arrest along with selective decreases in HDAC activity using a 15 µM concentration of SFN [10]. In addition, our lab observed SFN in combination with a green tea polyphenol (epigallocatechin gallate, EGCG) and found the compounds to work well in combination at decreasing colony forming potential and increasing apoptosis in chemo-resistant ovarian cancer cells. It was hypothesized and demonstrated that regulation of human telomerase reverse transcriptase (hTERT) and BCL-2 may serve as explanations for increases in apoptosis of ovarian cancer cells with the incorporation of combined SFN and EGCG [16]. 

In this current study we chose a much lower concentration of SFN to study in conjunction with WA, which may also have a significant impact on HDAC activity. To date there have been very limited studies implicating WA as an epigenetic modifier, and those that do have varying reports. Mirza and colleagues reported decreases in the transcript levels of *DNMT1*, *DNMT3A* and *DNMT3B* with the incorporation of WA, and use their findings to suggest that WA may have beneficial therapeutic effects against cancer through its ability to reverse changes in the epigenome [22]. In contrast, Szarc Vel Szic et al. were unable to show WA induced decreases in DNMTs [25]. In our study, we show variances between the different DNMTs with respect to the mRNA and protein levels with the treatment of WA and SFN. According to Dov Greenbaum and colleagues this is quite common, and what is found at the gene level is not a direct correlation of what may be found at the protein level. Along with there being several complex mechanisms involved in converting mRNA to protein, proteins also differ drastically in their half-lives [26]. Other studies have indicated this as well; in fact, Maier and colleagues wrote an extensive review that highlights several studies that have found weak correlations between gene and protein levels. Many studies have attempted to use changes in mRNA expression to gain understanding about potential changes in protein expression and the studies highlighted in this article demonstrate that gene expression is not always the best indicator for changes in the protein [27].

In an effort to gain clarity about what effects our chosen compounds have on breast cancer cells we sought to determine the role of WA on HDACs and DNMTs. Several studies have outlined the importance of DNMT1 and HDAC1 in tumor cell growth and development, hence the use of epigenetic inhibitors in the clinic [4,28,29]. One explanation for decreases in cellular viability induced by our compounds could be associated with the changes we observed in DNMT and HDAC expression. DNMT and HDAC activity assays were conducted to assess changes in these enzymes and to gain a general understanding of the effects of SFN and WA on the overall enzymatic activity of DNMTs and HDACs in breast cancer cells. Here we report significant decreases in overall DNMT and HDAC enzymatic activity in both MCF-7 ER (+) and MDA-MB-231 ER (−) breast cancer cells with the introduction of WA and SFN. To further analyze DNMTs and HDACs we assessed key epigenetic modifiers, DNMT1, DNMT3A, DNMT3B and HDAC1, and found decreases at both the mRNA and protein levels in one or both breast cancer cell lines. Our results indicate that combinatorial WA and SFN work extremely well in the inhibition of HDAC1 in both cell lines. An explanation for the lower significance in the HDAC activity assay in the MDA-MB-231 cells when comparing these results (Figure 6D) to the results in Figure 6B could be attributed to the fact that the activity assay is an assessment of overall enzymatic activity and there may be other HDACs that are contributing to our findings. The same can be noted with regard to the DNMTs (Figure 3 and Figure 4) as we show with the examination of DNMT3A and DNMT3B.

Our data demonstrate that combinatorial WA and SFN are effective in the inhibition of cell viability irrespective of ER status. Varying efficacy with respect to HDACs and DNMTs is to be expected due to the differing characteristics of each cell line. Previous studies show WA to be an inhibitor of ERα [30], while SFN is an activator [31]. MCF-7 cells are known to have a functional deletion of the caspase 3 gene [32]. This could also serve as an explanation for the variances between the MCF-7 and MDA-MB-231 cell lines. In Figure S2 we demonstrate that SFN + WA decreases cell viability and promotes apoptosis in T-47D cells, another ERα positive cell line without the functional deletion in the caspase-3 gene. These results indicate that the natural compounds used in this study are capable of killing ERα (+) breast cancer cells regardless of the presence or lack thereof of caspase 3. As it stands, these compounds could be competing with each other at the molecular level with regard to ER, which in turn is causing the differential effects in DNMTs and HDACs; however further study needs to be conducted to determine this. Although there were significant differences in HDAC and DNMT expression in these cells in response to WA and SFN, the combination of the two compounds resulted in even greater induction of apoptosis and less cell viability in both breast cancer cell lines. This implies that there are yet other factors that contribute to cell death initiated by these compounds. Several reports have shown that both WA and SFN are effective in the inhibition of pro-inflammatory cytokines, as well as the aberrant expression of epigenetic modifiers [11,13,33,34,35]. Moreover, Hahm and colleagues reported that WA-induced apoptosis was mediated through reactive oxygen species and Nagalingam et al. found that WA inhibited breast tumor formation in vivo through the activation of the extracellular signal-regulated kinases/ribosomal S6 kinase (ERK/RSK) axis, death receptor 5 (DR5) upregulation, and elevated nuclear accumulation of ETS domain-containing protein (Elk1) and C/EBP homologous protein (CHOP) in breast cancer [19,36]. 

We assessed the pro-apoptotic gene *BAX* and the anti-apoptotic gene *BCL-2* with combinatorial WA and SFN as well as singly administered SAHA and found there to be an inverse relationship in these treated breast cancer cells (Figure 9). Where *HDAC1* was decreased with our compounds in comparison to the FDA-approved chemotherapeutic SAHA (Figure 9A,D) we demonstrate an induction of *BAX* (Figure 9B,E) and a reduction of *BCL-2* with SFN + WA (Figure 9C,F). Interestingly, SFN + WA induced *BAX* expression to a greater extent than SAHA in MCF-7 cells. We recognize that many mechanisms may contribute to *BAX* induction. As seen in Figure 4A, SFN + WA affect DNMT1 expression greater than either compound alone in the ER (+) MCF-7 cells. Future studies may show that the combined effect of HDACs and DNMTs may be involved in *BAX* regulation in the MCF-7 cells. Nonetheless HDAC1 was down-regulated in both ER (+) and ER (−) cell lines. This finding supports the claim that HDAC1 regulation by combinatorial WA and SFN is responsible in part for induction of apoptosis in breast cancer cells.

In 2014 Xu and colleagues reported synergistic apoptotic effects with the combination of a synthetic HDAC inhibitor and DNMT inhibitor [37]. With the varying reports on WA being a DNMT inhibitor, we found merit in studying this compound. We confirm WA to be capable of inhibiting DNMTs to an extent and this compound shows synergy in reduction of cell viability when used in conjunction with SFN, a well-documented natural HDAC inhibitor. We hypothesize that the combined efficacy of these natural compounds on breast cancer cell death can be attributed in part through their impact on the epigenome. To begin establishing this we examined the clinically-approved HDAC inhibitor SAHA and found similar trends in comparison to combinatorial WA and SFN with the natural compounds being more effective in the promotion of the pro-apoptotic gene *BAX*, which is promising considering the numerous side effects associated with SAHA. This further confirms that the inhibition of both HDACs and DNMTs through the use of this novel combination of compounds (SFN + WA) may serve as a less harsh treatment option or preventive measure for breast cancer upon further study.

The current study has provided a basis of support behind the rationale to study WA and SFN in more depth with regard to specific epigenetic mechanisms. Our results support the role of combinatorial WA and SFN in the regulation of HDACs and also DNMTs, which are instrumental in a number of cancer developmental processes. Studies show WA to regulate mechanisms involved in the apoptotic pathway and our findings provide a framework to begin establishing epigenetic linkage of the combined WA and SFN with HDAC1 and cell cycle progression in cancer [20,33,36,38]. Future studies will focus on assessing more genes in association with epigenetic modifiers with the intent of providing a stronger association between HDAC1 and DNMTs and their regulation by combinatorial WA and SFN. In an effort to gain a better understanding of the epigenetic mechanisms involved in the changes induced by combinatorial WA and SFN, we intend to examine tumor suppressor genes that have been linked to epigenetic regulation by determining if there are any changes at the promoter region of the specified genes after treatment with these two compounds.

## 4. Materials and Methods

### 4.1. Cell Lines

The ERα (+) MCF-7 and ERα (−) MDA-MB-231 breast cancer cells were selected for this study. MCF10A human mammary epithelial cells were used as a non-cancerous control (ATCC, Manassas, VA, USA).

### 4.2. Chemicals

Withaferin A (≥95% pure) was purchased from Sigma-Aldrich (St. Louis, MO, USA), *R*,*S*-sulforaphane (≥98% pure) was acquired from LKT Laboratories (Minneapolis, MN, USA) and SAHA was purchased from Sigma-Aldrich (≥98% pure). Each compound was diluted in dimethyl sulfoxide (DMSO) and stored in stocks of 10 mmol/L at −20 °C.

### 4.3. Cell Culture and Treatment

MCF-7 and MDA-MB-231 were both cultured using Dulbecco's Modified Eagle's Medium DMEM 1× media in addition to 10% total volume of fetal bovine serum (FBS) (Atlanta Biologicals, Lawrenceville, GA, USA) and 1% total volume of 50× penicillin streptomycin (Corning Cellgro, Manassas, VA, USA). MCF10A cells were cultured using DMEM F12 media in addition to 5% Donor Horse Serum, 100 µL of 20 ng/ml EGF, 50 µL of 100 ng/mL cholera endotoxin, 100 µL of 0.05 µg/mL hydrocortisone, 0.292 g of 2 mmol/L l-Glutamine and 5 mL of 100 units/mL penicillin streptomycin. Cells were maintained in a humidified environment at 5% CO_2_ and 95% air at 37 °C. Cells were sub-cultured at approximately 90% confluency. After seeding, cells were allowed 24 h to adhere to plates after which they were treated over a one or three-day period with SFN, WA or both at the indicated concentrations. Treatments were replenished every 24 h with fresh media. DMSO was used as a vehicle control of which the maximum concentration was 1.2 µM. SFN and WA were stored as 10-mm stock solutions at −20 °C.

### 4.4. Cell Density Assay 

Approximately 200,000 cells were plated in 6-well plates. Upon the 24-h incubation period, treatments with WA and SFN were administered over a three-day period during which media was replaced accordingly. On day five after plating, cells were viewed under a microscope and images were taken at 100× or 40× magnification.

### 4.5. MTT Assay

Percent viability was determined by counting the number of viable cells in each well via the uptake of tetrazolium, 3-(4,5-dimethylthiazol-2-yl)-diphenyl tetrazolium bromide (MTT) (Sigma-Aldrich). The living cells cause a dark purple color to appear due to a formazan reaction initiated by the mitochondrial enzymes of the cells. Approximately 2000 cells were seeded in triplicate and allowed to incubate for 24 h to adhere to the 96-well plates. The cells were treated over a one or three-day period as described above. On day three or day five after plating, 50 µL of MTT (1 mg/mL) dissolved from 5 g/L in phosphate-buffered saline (PBS) wash buffer was added and allowed to incubate at 37 °C for 3 h after which the MTT reagent was removed and DMSO was added to each well. A microplate reader (model 680, Bio-Rad, Hercules, CA, USA) with the absorbance set to read at 595 nm was then used to obtain the values that determined % viability.

### 4.6. RNA Isolation

RNA was extracted using the RNeasy kit from Qiagen (Valencia, CA, USA) according to the manufacturer’s instructions. 

### 4.7. Protein Extraction 

Radioimmunoprecipitation (RIPA) Lysis Buffer from Upstate Biotechnology (Charlottesville, VA, USA) was used to prepare protein extracts according to the manufacturer’s protocol.

### 4.8. Quantitative Real Time PCR (qRT-PCR)

qRT-PCR was used to determine the expression of specific genes of interest. RNA was reverse transcribed to cDNA using the cDNA synthesis kit from Bio-Rad. PCR reactions were completed in triplicate using 1 µL of cDNA for each sample. Both forward and reverse primers (1 µL) for the gene of interest were used along with 5 µL of iTaq SYBR green from Bio-Rad and 2 µL of nuclease free water for a total volume of 10 µL. Once samples were prepared they were placed in the CFX Connect Real Time System from Bio-Rad upon which the three-step amplification protocol was selected. Thermal cycling was initiated at 94 °C for 4 min followed by 35 cycles of PCR (94 °C, 15 s; 60 °C, 30 s; 72 °C, 30 s). GAPDH was used as an endogenous control in order to calculate fold change using the ΔΔ*C*_q_ method described by Chen et al [17]. Primers were purchased from Integrated DNA Technologies (IDT, Coralville, IA, USA), and sequences are listed in Table 2. 

### 4.9. Annexin V Apoptosis Assay FACS

The induction of apoptosis in breast cancer cells via WA and SFN was quantitatively determined using flow cytometry and the Annexin V—conjugated Alexafluor 488 (Alexa 488) Apoptosis Vybrant Assay Kit (Life Technologies, Carsbald, CA, USA). After treatment, cells were harvested using the digestive enzyme trypsin. Upon detachment, cell pellets were collected via centrifugation. PBS wash buffer was used to wash pelleted cells twice, and after washing, cells were incubated with Alexa488 and propidium iodide (PI) for cellular staining in annexin binding buffer for 10 min in the dark at room temperature. The stained cells were analyzed by fluorescence-activated cell sorting (FACS) by using a FACS-Caliber instrument (BD Biosciences, San Jose, CA, USA) equipped with Cell Quest 3.3 software (BD Biosciences, San Jose, CA, USA).

### 4.10. Western Blot Analysis

Protein expression was determined with the use of western blotting. Protein extracts were prepared by RIPA Lysis Buffer as mentioned previously. Bradford assays were performed to determine the protein concentration (Bio-Rad Protein Assay, Bio-Rad). The protein was loaded onto a 4–15% premade Tris-HCl gel from Bio-Rad, and separated by electrophoresis at 200 V until the dye ran off the gel. Separated proteins were then transferred to nitrocellulose membrane using the Trans Turbo Blot from Bio-Rad. Membranes were then blocked in 5% dry milk in Tris-buffered saline (TBS) solution with 1% Tween (TBST) using the Millipore SnapID (Billerica, MA, USA). Primary antibody incubations were carried out at room temperature and membranes were washed four times with 30 mL of TBST before probing with secondary antibody for 1 h followed by four more washes. Immunoreactive bands were visualized using an enhanced chemiluminescence detection system (Bio-Rad). Santa Cruz Biotechnology (Dallas, TX, USA) and Cell Signaling Technology (Danvers, MA, USA) were the suppliers of the selected antibodies.

### 4.11. DNMTs Activity Assay

After treatment with WA and SFN accordingly, nuclear extracts were prepared using the EpiQuik nuclear extraction kit from EpiGenTek (OP-0002-1). DNMT activity was determined via the EpiQuik DNA Methyltransferase Activity/Inhibition Colorimetric Assay Kit (P-3009) following the manufacturer’s procedures (Farmingdale, NY, USA).

### 4.12. HDACs Activity Assay

Nuclear extracts were prepared as mentioned above, and the EpiQuik HDAC Activity/Inhibition Colorimetric Assay Kit (P-4002) was used. The assay was performed according to the provided protocol from EpiGenTek (Farmingdale, NY, USA).

### 4.13. CompuSyn

The CompuSyn version 1.0 software (Available online: http://www.combosyn.com/) used to determine synergism of the combinatorial WA and SFN. A combination index (CI) value greater than 1 denotes antagonism, a value below 1 indicates synergism and a value at one indicates an additive effect of the compounds being assessed [24,39].

### 4.14. Statistical Analysis

Error bars represent standard error of the mean (SEM). Each assay was completed in triplicate culturing experiments with three or four technical replicates. The student’s *t*-test was used to determine significance.

## 5. Conclusions

In summary, WA and SFN are two compounds that have been shown to be effective inhibitors of cancer cell growth; however, the literature is limited with respect to WA and its regulatory roles on key epigenetic modifiers. Prior studies have also not yet addressed the effects of either of these compounds in conjunction with one another. We report greater efficacy of these compounds in combination with regard to breast cancer cell death and down-regulation of overexpressed HDAC1, DNMT3A and DNMT3B. We believe that further study of combinatorial WA and SFN may have translational significance through their potential to serve as ideal candidates for prevention of breast cancer-related fatality.

## Figures and Tables

**Table 1:** CompuSyn data of MTT values indicate combinatorial synergy in MCF-7 cells. CI: combination index.

Cell Lines	SFN Dose	WA Dose	Average CI
MCF-7	5.0 µM	1.0 µM	0.715503
MDA-MB-231	5.0 µM	1.0 µM	1.000525

**Table 2:** qRT-PCR primer sequences.

Forawrd Primer Sequences	Reverse Primer Sequences
DNMT1 Sense: 5′-AACCTTCACCTAGCCCCAG-3′	DNMT1 Anti-sense: 5′-CTCATCCGATTTGGCTCTTCA-3′
DNMT3A Sense: 5′-TATTGATGAGCGCACAAGGC-3′	DNMT3A Anti-sense: 5′-GGGTGTTCCAGGGTAACATTGAG-3′
DNMT3B Sense: 5′-TGGTACATGGCTTTTCGATAGGA-3′	DNMT3B Anti-sense: 5′-GGCAAGTTCTCCGAGGTCTCTG-3′
HDAC1 Sense: 5′-CTGTCCGGTATTTGATGGCT-3′	HDAC1 Anti-sense: 5′-CACGAACTCCACACACTTGG-3′
BAX Sense: 5′-TGG AGCTGCAGAGGATGATTG-3′	BAX Anti-sense: 5′-GAAGTTGCCGTCAGAAAACATG-3′
BCL-2 Sense: 5′-CATGCTGGGGCCGTACAG-3′	BCL-2 Anti-sense: 5′-GAACCGGCACCTGCACAC-3′
GAPDH Sense: 5′-TGCACCACCAACTGCTTAGC-3′	GAPDH Anti-Sense: 5′-GGCATGGACTGTGGTCATGAG-3′
Caspase 3 Sense: 5′-TTAATAAAGGTATCCATGGAGAACACT-3′	Caspase 3 Anti-Sense: 5′-TTAGTGATAAAAA TAGAGTTCTTTTGTGAG-3′

Primers were purchased from Integrated DNA Technologies (IDT) using the indicated primer sequences.

## Supplementary Materials

Supplementary materials can be found at www.mdpi.com/1422-0067/18/5/1092/s1.

## References

[1] Allis C.D., Jenuwein T. (2016). The molecular hallmarks of epigenetic control. Nat. Rev. Genet..

[2] Gryder B.E., Sodji Q.H., Oyelere A.K. (2012). Targeted cancer therapy: Giving histone deacetylase inhibitors all they need to succeed. Future Med. Chem..

[3] Jung Y., Park J., Kim T.Y., Park J.-H., Jong H.-S., Im S.-A., Robertson K.D., Bang Y.-J., Kim T.-Y. (2007). Potential advantages of DNA methyltransferase 1 (DNMT1)-targeted inhibition for cancer therapy. J. Mol. Med..

[4] Bashtrykov P., Jeltsch A. (2015). DNMT1-associated DNA methylation changes in cancer. Cell Cycle.

[5] Mann B.S., Johnson J.R., Cohen M.H., Justice R., Pazdur R. (2007). FDA approval summary: Vorinostat for treatment of advanced primary cutaneous T-cell lymphoma. Oncologist.

[6] DeSantis C.E., Fedewa S.A., Goding Sauer A., Kramer J.L., Smith R.A., Jemal A. (2016). Breast cancer statistics, 2015: Convergence of incidence rates between black and white women. CA Cancer J. Clin..

[7] Esmaeili M.A. (2016). Combination of siRNA-directed gene silencing with epigallocatechin-3-gallate (EGCG) reverses drug resistance in human breast cancer cells. J. Chem. Biol..

[8] Li Y., Meeran S.M., Patel S.N., Chen H., Hardy T.M., Tollefsbol T.O. (2013). Epigenetic reactivation of estrogen receptor-α (ERα) by genistein enhances hormonal therapy sensitivity in ERα-negative breast cancer. Mol. Cancer.

[9] Cheng Y.M., Tsai C.C., Hsu Y.C. (2016). Sulforaphane, a dietary isothiocyanate, induces G2/M arrest in Cervical cancer cells through CyclinB1 downregulation and GADD45β/CDC2 association. Int. J. Mol. Sci..

[10] Clarke J.D., Hsu A., Yu Z., Dashwood R.H., Ho E. (2011). Differential effects of sulforaphane on histone deacetylases, cell cycle arrest and apoptosis in normal prostate cells versus hyperplastic and cancerous prostate cells. Mol. Nutr. Food Res..

[11] Sharma C., Sadrieh L., Priyani A., Ahmed M., Hassan A.H., Hussain A. (2011). Anti-carcinogenic effects of sulforaphane in association with its apoptosis-inducing and anti-inflammatory properties in human cervical cancer cells. Cancer Epidemiol..

[12] Su Z.-Y., Zhang C., Lee J.H., Shu L., Wu T.-Y., Khor T.O., Conney A.H., Lu Y.-P., Kong A.-N.T. (2014). Requirement and epigenetics reprogramming of Nrf2 in suppression of tumor promoter TPA-induced mouse skin cell transformation by sulforaphane. Cancer Prev. Res..

[13] Tomczyk J., Olejnik A. (2010). Sulforaphane—A possible agent in prevention and therapy of cancer. Postepy Hig. Med. Dosw..

[14] Royston K.J., Tollefsbol T.O. (2015). The epigenetic impact of cruciferous vegetables on cancer prevention. Curr. Pharmacol. Rep..

[15] Ho E., Clarke J.D., Dashwood R.H. (2009). Dietary sulforaphane, a histone deacetylase inhibitor for cancer prevention. J. Nutr..

[16] Chen H., Landen C.N., Li Y., Alvarez R.D., Tollefsbol T.O. (2013). Epigallocatechin gallate and sulforaphane combination treatment induce apoptosis in paclitaxel-resistant ovarian cancer cells through hTERT and Bcl-2 down-regulation. Exp. Cell Res..

[17] Chen H., Landen C.N., Li Y., Alvarez R.D., Tollefsbol T.O. (2013). Enhancement of cisplatin-mediated apoptosis in ovarian cancer cells through potentiating G2/M arrest and p21 upregulation by combinatorial epigallocatechin gallate and sulforaphane. J. Oncol..

[18] Fong M.Y., Jin S., Rane M., Singh R.K., Gupta R., Kakar S.S. (2012). Withaferin A synergizes the therapeutic effect of doxorubicin through ROS-mediated autophagy in ovarian cancer. PLoS ONE.

[19] Hahm E.-R., Moura M.B., Kelley E.E., van Houten B., Shiva S., Singh S.V. (2011). Withaferin A-induced apoptosis in human breast cancer cells is mediated by reactive oxygen species. PLoS ONE.

[20] Hahm E.R., Singh S.V. (2013). Withaferin A-induced apoptosis in human breast cancer cells is associated with suppression of inhibitor of apoptosis family protein expression. Cancer Lett..

[21] Kim S.-H., Hahm E.-R., Arlotti J.A., Samanta S.K., Moura M.B., Thorne S.H., Shuai Y., Anderson C.J., White A.G., Lokshin A. (2016). Withaferin A inhibits in vivo growth of breast cancer cells accelerated by Notch2 knockdown. Breast Cancer Res. Treat..

[22] Mirza S., Sharma G., Parshad R., Gupta S.D., Pandya P., Ralhan R. (2013). Expression of DNA methyltransferases in breast cancer patients and to analyze the effect of natural compounds on DNA methyltransferases and associated proteins. J. Breast Cancer.

[23] Mohan R., Hammers H., Bargagna-mohan P., Zhan X., Herbstritt C., Ruiz A., Zhang L., Hanson A., Conner B., Rougas J. (2004). Withaferin A is a potent inhibitor of angiogenesis. Angiogenesis.

[24] Chou T.C. (2008). Preclinical versus clinical drug combination studies. Leuk. Lymphoma.

[25] Szarc vel Szic K., Op de Beeck K., Ratman D., Wouters A., Beck I.M., Declerck K., Heyninck K., Fransen E., Bracke M., de Bosscher K. (2014). Pharmacological levels of Withaferin A (Withania somnifera) trigger clinically relevant anticancer effects specific to triple negative breast cancer cells. PLoS ONE.

[26] Greenbaum D., Colangelo C., Williams K., Gerstein M. (2003). Comparing protein abundance and mRNA expression levels on a genomic scale. Genome Biol..

[27] Maier T., Guell M., Serrano L. (2009). Correlation of mRNA and protein in complex biological samples. FEBS Lett..

[28] Pathania R., Ramachandran S., Elangovan S., Padia R., Yang P., Cinghu S., Veeranan-Karmegam R., Arjunan P., Gnana-Prakasam J.P., Sadanand F. (2015). DNMT1 is essential for mammary and cancer stem cell maintenance and tumorigenesis. Nat. Commun..

[29] Senese S., Zaragoza K., Minardi S., Muradore I., Ronzoni S., Passafaro A., Bernard L., Draetta G.F., Alcalay M., Seiser C. (2007). Role for histone deacetylase 1 in human tumor cell proliferation. Mol. Cell Biol..

[30] Hahm E.R., Lee J., Huang Y., Singh S.V. (2011). Withaferin a suppresses estrogen receptor-α expression in human breast cancer cells. Mol. Carcinog..

[31] Meeran S.M., Patel S.N., Li Y., Shukla S., Tollefsbol T.O. (2012). Bioactive dietary supplements reactivate ER expression in ER-negative breast cancer cells by active chromatin modifications. PLoS ONE.

[32] Liang Y., Yan C., Schor N.F. (2001). Apoptosis in the absence of caspase 3. Oncogene.

[33] Um H.J., Min K.J., Kim D.E., Kwon T.K. (2012). Withaferin A inhibits JAK/STAT3 signaling and induces apoptosis of human renal carcinoma Caki cells. Biochem. Biophys. Res. Commun..

[34] Abbas A., Hall J.A., Patterson W.L., Ho E., Hsu A., Al-Mulla F., Georgel P.T. (2016). Sulforaphane modulates telomerase activity via epigenetic regulation in prostate cancer cell lines. Biochem. Cell Biol..

[35] Meeran S.M., Patel S.N., Tollefsbol T.O. (2010). Sulforaphane causes epigenetic repression of hTERT expression in human breast cancer cell lines. PLoS ONE.

[36] Nagalingam A., Kuppusamy P., Singh S.V., Sharma D., Saxena N.K. (2014). Mechanistic elucidation of the antitumor properties of withaferin a in breast cancer. Cancer Res..

[37] Xu S., Ren J., Chen H.B., Wang Y., Liu Q., Zhang R., Jiang S.W., Li J. (2014). Cytostatic and apoptotic effects of DNMT and HDAC inhibitors in endometrial cancer cells. Curr. Pharm. Des..

[38] Zhang X., Samadi A.K., Roby K.F., Timmermann B., Cohen M.S. (2012). Inhibition of cell growth and induction of apoptosis in ovarian carcinoma cell lines CaOV3 and SKOV3 by natural withanolide Withaferin A. Gynecol. Oncol..

[39] Kala R., Shah H.N., Martin S.L., Tollefsbol T.O. (2015). Epigenetic-based combinatorial resveratrol and pterostilbene alters DNA damage response by affecting SIRT1 and DNMT enzyme expression, including SIRT1-dependent gamma-H2AX and telomerase regulation in triple-negative breast cancer. BMC Cancer.

