# Supplementary material for: A Novel Combination of Withaferin A and Sulforaphane Inhibits Epigenetic Machinery, Cellular Viability and Induces Apoptosis of Breast Cancer Cells
Source: Int J Mol Sci. 2017 May 19;18(5):1092. doi: 10.3390/ijms18051092 (PMC5455001; doi:10.3390/ijms18051092)
Supplement: Supplementary file 1 [file ijms-18-01092-s001.pdf]

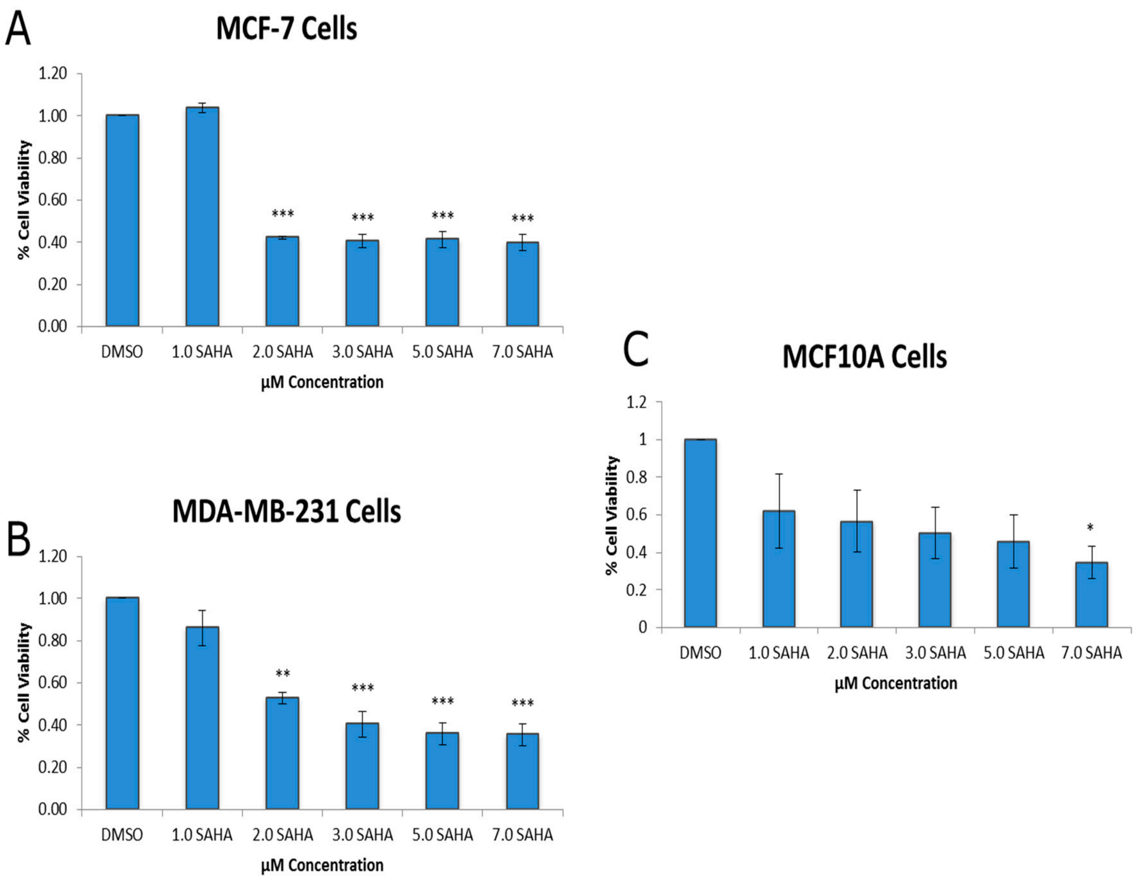

Figure S1. FDA-approved HDAC inhibitor SAHA decreases cellular viability in MCF-7 and MDA-MB-231 breast cancer cells. (**A**) MTT assay of MCF-7 cells indicates decreases in cell viability at increasing concentrations of SAHA. (**B**) MDA-MB-231 cells show decreases in viability at increasing concentrations of SAHA. (**C**) The non-cancerous MCF10A cells show statistically insignificant decreases in cell viability except for the relatively high concentration of 7 µM SAHA. ($n = 3$: SEM, * $p < 0.05$, ** $p < 0.01$, *** $p < 0.001$).

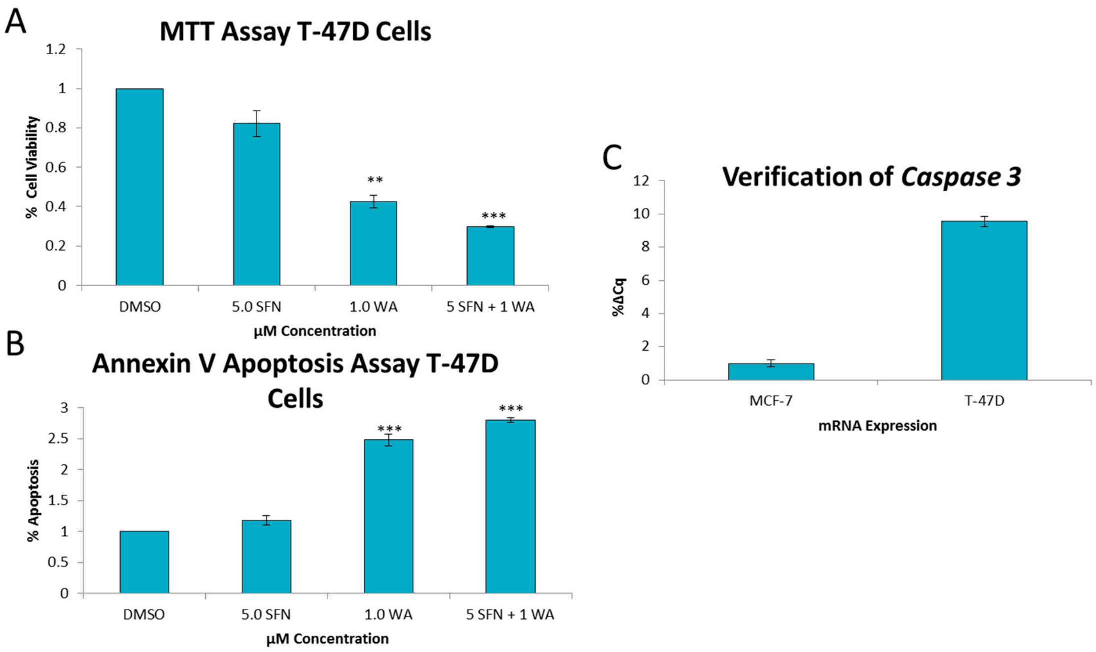

Figure S2. SFN and WA promote cell death in T-47D breast cancer cells. (**A**) MTT assay of T-47D cells indicates decreases in cell viability with the incorporation of the indicated compounds after 3 days. (**B**) FACS analysis demonstrates an increase in apoptosis caused by combinatorial WA and SFN after 3 days. (**C**) Cells were treated for 3 days with DMSO. qRT-PCR verifies that the caspase 3 gene is expressed in T-47D breast cancer cells (n = 3: SEM, ** $p < 0.01$, *** $p < 0.001$).
